# Development of Apremilast Nanoemulsion-Loaded Chitosan Gels: In Vitro Evaluations and Anti-Inflammatory and Wound Healing Studies on a Rat Model

**DOI:** 10.3390/gels8050253

**Published:** 2022-04-20

**Authors:** Mohammed Muqtader Ahmed, Md. Khalid Anwer, Farhat Fatima, Amer S. Alali, Mohd Abul Kalam, Ameeduzzafar Zafar, Sultan Alshehri, Mohammed M. Ghoneim

**Affiliations:** 1 Department of Pharmaceutics, College of Pharmacy, Prince Sattam Bin Abdulaziz University, P.O. Box 173, Al-Kharj 11942, Saudi Arabia; m.anwer@psau.edu.sa (M.K.A.); f.soherwardi@psau.edu.sa (F.F.); a.alali@psau.edu.sa (A.S.A.); 2 Nanobiotechnology Unit, Department of Pharmaceutics, College of Pharmacy, King Saud University, P.O. Box 2457, Riyadh 11451, Saudi Arabia; makalam@ksu.edu.sa; 3 Department of Pharmaceutics, College of Pharmacy, King Saud University, Riyadh 11451, Saudi Arabia; salshehri1@ksu.edu.sa; 4 Department of Pharmaceutics, College of Pharmacy, Jouf University, Aljouf Region, Sakaka 72341, Saudi Arabia; azafar@ju.edu.sa; 5 Department of Pharmacy Practice, College of Pharmacy, Almaarefa University, Ad Diriyah 13713, Saudi Arabia; mghoneim@mcst.edu.sa

**Keywords:** apremilast, nanoemulsion, chitosan, gels, stability, anti-inflammatory, wound healing

## Abstract

Apremilast (APL) has profound anti-inflammatory and wound healing activity, alongside other dermal care. This study aims to develop APL-loaded NEs (ANE1-ANE5) using eucalyptus oil (EO) as the oil and Tween-80 and transcutol-HP (THP) as a surfactant and co-surfactant, respectively. The prepared NEs were then evaluated based on mean droplet size (12.63 ± 1.2 nm), PDI (0.269 ± 0.012), ZP (−23.00 ± 5.86), RI (1.315 ± 0.02), and %T (99.89 ± 0.38) and ANE4 was optimized. Further, optimized NEs (ANE4) were incorporated into chitosan gel (2%, *w/v*). The developed ANE4-loaded chitosan gel was then evaluated for pH, spreadability, in vitro diffusion, and wound healing and anti-inflammatory studies. Moreover, in vivo studies denoted improved anti-inflammatory and wound healing activity and represented a decrease in wound size percentage (99.68 ± 0.345%) for the APNE2 gel test compared to a negative control (86.48 ± 0.87%) and standard control (92.82 ± 0.34%). Thus, the formulation of ANE4-loaded chitosan gels is an efficient topical treatment strategy for inflammatory and wound healing conditions.

## 1. Introduction

Apremilast (APL) is a selective PDE4 inhibitor indicated for plaque psoriasis and psoriatic arthritis [1]. Its recommended dosage regimen is 10 mg as a starting dose twice daily followed by 30 mg in five days and 30 mg taken thereafter. APL is considered a small molecule with a molecular weight of 460.50 with a half-life of 6–9 h. It is a pale yellow powder that is insoluble in water and has a BCS-IV classification [2]. APL acts as a phosphodiesterase 4 (PDE4) inhibitor that suppresses the cyclic adenosine monophosphate (cAMP) degradation to AMP in cells. The increased intracellular secondary messenger cAMP, due to the activation of adenylyl cyclase, influences a wide array of cellular functions; activates transcription factor nuclear factor-kappa B (NF-κB) and the expression of pro-inflammatory cytokines such as IL-23, tumor necrosis factor-α (TNF-α), and interferon-gamma (IFN-γ); and regulates anti-inflammatory interleukins [3,4]. Briefly, APL increases intracellular cAMP levels and then modulates the production of inflammatory mediators indirectly [5]. Inflammation may also be instigated by the derivative of arachidonic acid and cyclooxygenase; APL inhibits cyclooxygenase and attenuates inflammation [6]. Chronic inflammatory conditions lead to cell death and mutation in the cell organelles, initiating uncontrolled cell growth—cancer [7]. World Health Organization (WHO) data and a retrospective study show that diabetic foot ulcers in patients represent the greatest threat to public health and the national health budget due to the steady rise in the prevalence of such conditions [8]. The use of APL isolated from botanicals has been reported to exhibit a potential wound healing effect [9]. APL also elicits enhanced wound healing and tissue repair in animals with induced diabetes. Wound healing is a process involving four critical stages: hemostasis, inflammation, proliferation, and remodeling [10].

APL is administered orally but has exhibited some notable side effects, such as first pass metabolism, gastrointestinal side effects, and a longer duration of treatment, which impairs patient compliance. Therefore, a need arises to develop a topical formulation to bypass these side effects. The topical route of drug administration provides a simple and painless option in the treatment of dermatological illnesses [11,12]. However, the major challenge for topical formulation is the restriction of the transdermal diffusion of the drug via the stratum corneum, which limits the intended therapeutic effect of the drug. As a result, the incorporation of APL into nanocarrier-based drug delivery systems such as NEs might be employed to increase its permeability, hence potentially increasing the local deposition of the drug at the desired site and, as a result, achieving local anti-inflammatory activity and wound healing [13,14].

Nanoemulsion (NEs) can accommodate both hydrophilic and lipophilic drugs [15,16]. Most NEs have been developed by ingredients listed as “Generally Recognized as Safe” (GRAS) by the FDA [17]. Generally, high-energy emulsification and low-energy emulsification are broadly used to develop NEs. NEs are isotropic dispersed systems composed of oil, surfactants, and water. They are thermodynamically stable dispersions of small elastic globules with a nanoscale range of 20–500 nm [18]. Chitosan-based gels have gained attention in recent years, especially in formulation development. Chitosan is a unique biopolymer having a low toxicity, degradability, and non-immunogenic properties [19,20,21].

Therefore, the objective of this study was to develop APL nanoemulsion-loaded chitosan gels for the anti-inflammatory and wound healing activity. The developed NEs were optimized based on the physicochemical evaluations. After that, the optimized NEs were incorporated into chitosan gels and evaluated for anti-inflammatory and wound healing activity on rats.

## 2. Results and Discussion

### 2.1. Determination of APL Solubility in Oils, Surfactants, and Co-Surfactants

**Figure 1:**
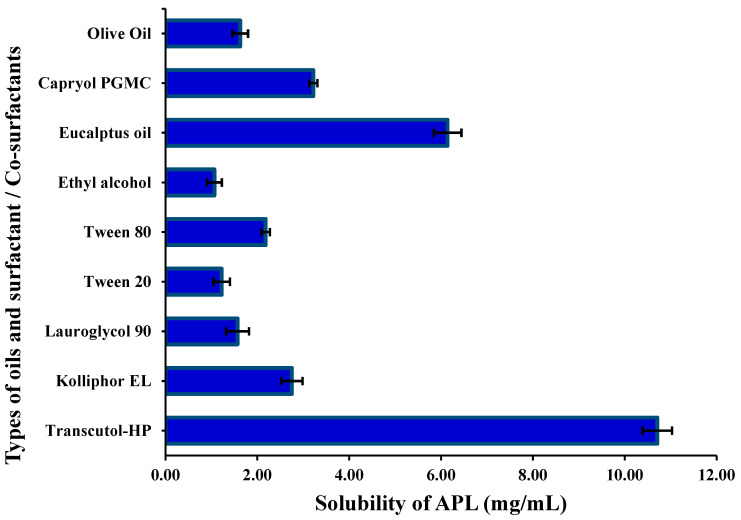
Phase solubility of apremilast in various oils, surfactant, and co-surfactants.

The solubility of APL was determined in some selected pure oils, surfactants, and co-surfactants and is represented in Figure 1. The highest solubility of APL was found in eucalyptus oil, Tween 80, and THP at 6.14 ± 0.31 mg/mL, 2.18 ± 0.09 mg/mL, and 10.71 ± 0.32 mg/mL, respectively. However, APL is a hydrophobic drug, and thus the amount of the drug incorporated in NEs is essential. Drugs with a poor solubility require more oil to be incorporated, which will increase the formulation cost, so blends of a surfactant and co-surfactant are used to produce stable and cost-effective NEs [22].

### 2.2. Pseudo-Ternary Phase Diagram

**Figure 2:**
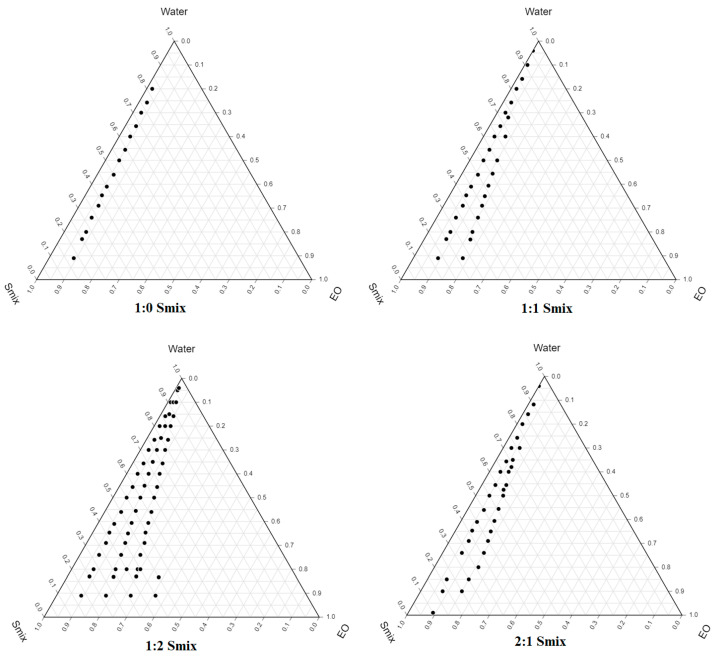
Pseudo-ternary phase diagrams of eucalyptus oil (EO), Tween 80: THP (Smix) and water.

The pseudo-ternary phase diagrams were constructed using EO (oil), Smix (Tween-80 and THP), and water (Figure 2). When Tween-80 was utilized without THP (Smix ratio 1:0), it was observed that Tween-80 alone is insufficient to generate stable NEs. The zone of NEs area in the phase diagram grew immensely when surfactants and co-surfactants were used in equal proportions (Smix ratio 1:1) compared to Smix ratio 1:0 [23,24]. THP reduces the interfacial tension and makes the interfacial film flexible enough to take on the various curvatures necessary to create NEs over a wide variety of compositions. The zone of NEs area was increased when the content of THP as a co-surfactant was increased (Smix ratio 1:2) (Figure 2). The increase in the NEs area is possibly due to a high concentration of co-surfactant, which increases the solubilization capacity of NEs.

### 2.3. Physicochemical Properties of Developed APL-Loaded NEs

**Figure 3:**
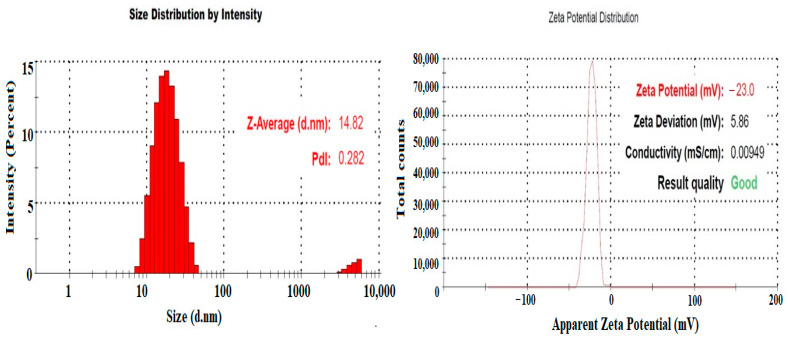
Mean droplet size and zeta potential of APL-loaded NE (ANE4).

Mean droplet size, PDI, and ZP of APL loaded NEs (ANE1-ANE5) were measured in the range of 12.63 ± 1.2 to 196.6 ± 7.4 nm, 0.078 ± 0.004 to 0.340 ± 0.003, and −1.35 ± 0.00 to −23.00 ± 5.86 mV, respectively (Table 1). Among the prepared NEs (ANE1-ANE5), ANE4 had lowest droplet size (12.63 ± 1.2 nm) with PDI (0.078 ± 0.004) and highest ZP (−23.00 ± 5.86 mV) (Figure 3). The highest ZP values of NE4 (−23.00 ± 5.86 mV) indicates a stable NEs among other formula. Negative charged ZP of the NEs is probably due to the anionic group of the oils, and Tween-80 and THP present in the Smix [25]. There average values of RI’s and % T for NEs (ANE1-ANE5) were measured in the range 1.311 ± 0.05 − 1.421 ± 0.05 and 99.89 ± 0.38 − 96.75 ± 0.16%, respectively. The lowest RI’s value for ANE1 (1.311 ± 0.05) was measured, probably due to lowest content of oil in the NE [26]. The highest %T was observed for NE4, indicating a transparent formulation. These values are closer to 100% transmittance exhibiting the clear, transparent, and isotropic NE.

### 2.4. Thermodynamic Stability Study

Thermodynamic stability was accessed to exclude the possibility of metastable/unstable NEs. All developed APL-loaded NEs (ANE1-ANE5) were observed thermodynamically stable after centrifugation, heating and cooling and freeze-thaw cycles. Results of thermodynamic stability reflects long shelf life to the ANE4, relatively as compared to other NEs. Among all developed APL-loaded NEs (ANE1-ANE5), ANE4 was found thermodynamically stable. Therefore, based on mean droplet size, PDI, ZP, RI, %T and thermal stability data, ANE4 was found optimum and further subjected for incorporation into chitosan gel.

### 2.5. TEM Images

**Figure 4:**
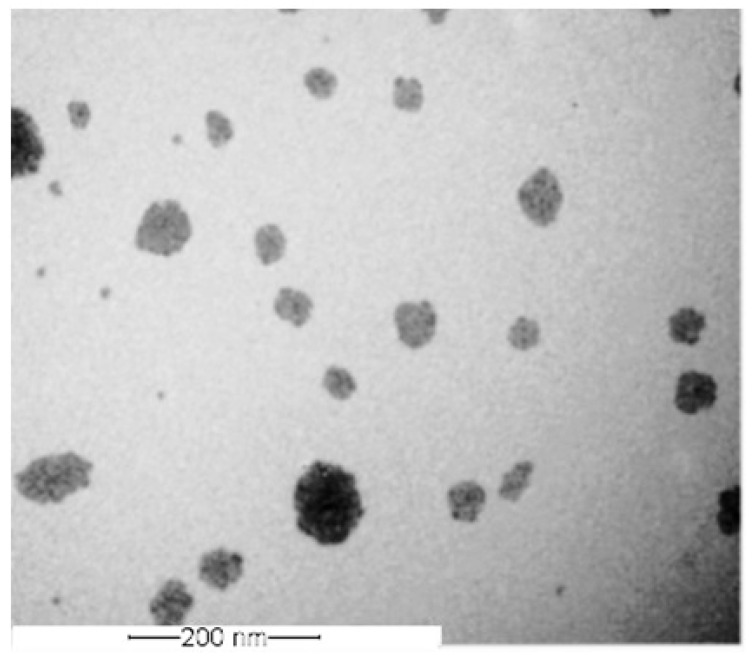
TEM images of optimized NEs (ANE4).

The optimized NEs (ANE4), microphotograph represents spherical shape with rough surface and size was found in the nano-range. The rough surface of the nanoemulsion might be due to sample preparation during TEM studies (Figure 4). The size can be correlated with the results of globule size showed by photon correlation spectroscopy provided by Zetasizer.

### 2.6. Drug–Excipient Interaction FTIR Study

**Figure 5:**
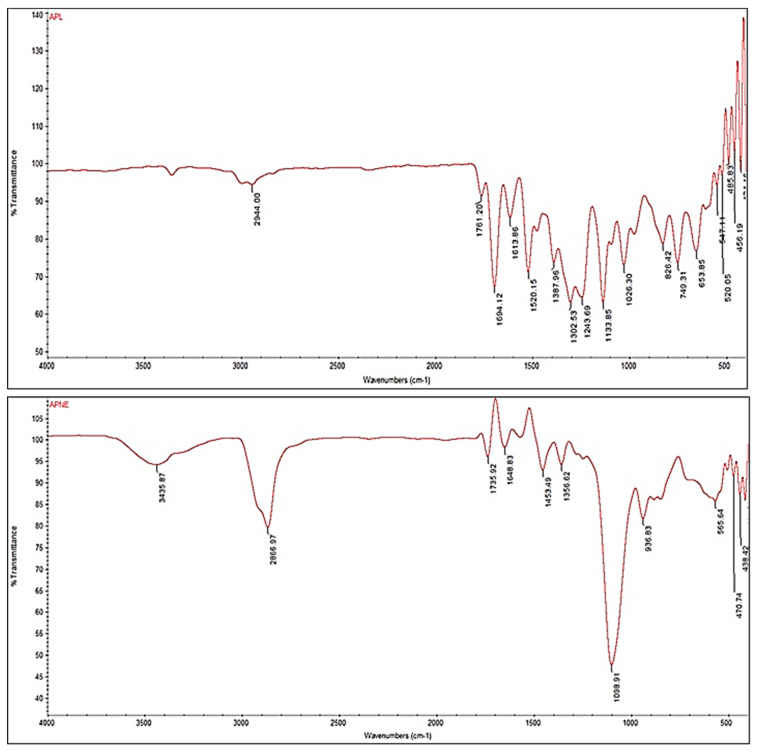
FTIR spectrums of pure APL and APL-loaded NEs (ANE4).

FTIR spectra of pure APL exhibited prominent peaks at 2944 cm^−1^ (C-H stretching), 1694 cm^−1^ (C=O bending), 1520 cm^−1^ (-CONH-). Peaks at 1387.96 cm^−1^, 1134 cm^−1^, 1026 cm^−1^ for C-C ring stretching, C-O bending, N stretching, respectively [27]. Moreover, APL-loaded NEs (ANE4), showed peaks at 2867 cm^−1^, 1736 cm^−1^, 1649 cm^−1^,1453 cm^−1^, 1357 cm^−1^, 1099 cm^−1^, 937 cm^−1^ corresponding to the APL with additional peaks at 566 cm^−1^ (C–C ring deformation), 471 cm^−1^ and 439 cm^−1^ for C–N ring deformation and N–O deformation, respectively (Figure 5). There is reduction in peak intensity in NEs (ANE4) could be seen due to entrapment of drug in globules. These peaks clearly indicates that APL was compatible with excipients used in the preparation of NEs.

### 2.7. Evaluation of APL-Loaded NEs Chitosan Based Gel

The pH measurement govern skin compatibility with developed gel, suitable for topical application and free from allergic reaction and skin irritation. The pH values of ANE4-loaded gel was noted to be 6.8 ± 1.2, which is optimum for dermal application. The success of topical gel depends on the spreadability, it’s an important parameter on which therapeutic efficacy relies. A small shear is required for uniform spreadability of gels to the skin [28]. Spreadability of the developed NE4-loaded gels exhibited the optimal spreadability over the plexy glass plates the area of formulation spread was observed as be 8.6 cm, suggesting practically excellent spreadability.

### 2.8. Diffusion Study and Release Kinetics Models

**Figure 6:**
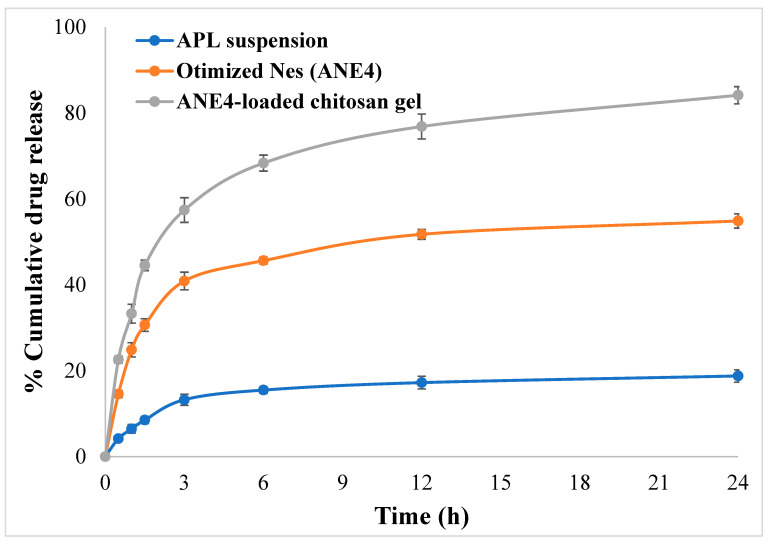
Cumulative drug release from pure APL suspension, optimized NEs (ANE4) APNE4-loaded gel.

Comparative in-vitro diffusion study was performed for pure drug (APL), optimized NEs (NE4) and ANE4-loased gel (Figure 6). Cumulative percentage drug release was found 18.79% from pure APL suspension, 54.88% and 84.17% from optimized NEs (ANE4) and ANE4-loaded gel after 24 h, respectively. An improved drug release was observed from developed topical gel due to its nanosize carrier, which could facilitate drug penetration across the dermal layer and improve therapeutic concentration at the local site [29]. The release kinetics was also determined by fitting the data into different kinetics models. Table 2 showed data of different kinetic models with highest correlation coefficient value (R^2^ = 0.9242) for Korsmeyer–Peppas followed by the Higuchi model (R^2^ = 0.8572), First-order (R^2^ = 0.8265), and Zero order (R^2^ = 0.6162). Among all fitted models, APNE4-loaded chitosan gels release pattern was best fitted to Korsmeyers-Peppas model with release exponent (*n*) ≤ 0.45 indicating Fickian diffusion (case I diffusional) release mechanism. Drug release improved as NEs containing oil, surfactant and co-surfactant was incorporated into chitosan gel. Drug was released from NE4-loade chitosan gel by erosion mechanism [30,31]. Free drug-loaded NEs droplets could be easily permeated at a higher rate into the skin layers.

### 2.9. Stability Study

Cumulative % drug release at the 24th h was found to be 82.24% from ANE4-loaded gel, and after the shelf-life study period, it was found to be 81.41 ± 2.38%. The similarity factor was found to be 51.42, and drug content 97.16 ± 2.65% in the ANE4-loaded gel formulation. Insignificant variation in these parameters indicates developed gel was stable.

### 2.10. Anti-Inflammatory Studies

**Figure 7:**
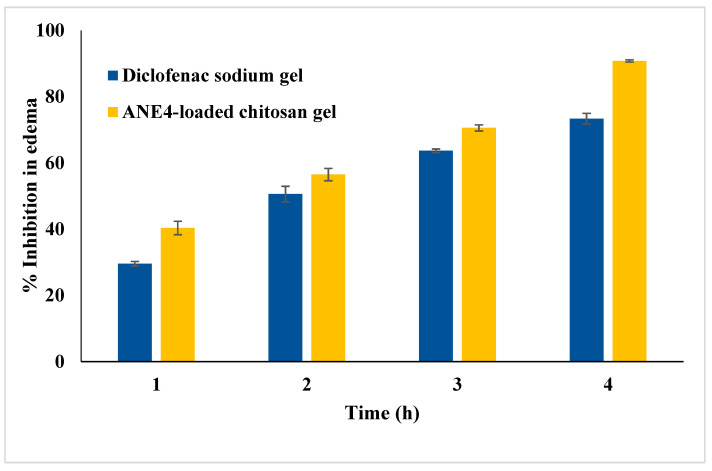
Comparative anti-inflammatory activity of APNE4-loaded gel and Diclofenac sodium gel.

The anti-inflammatory efficacy of ANE4-loaded chitosan gel was compared with Diclofenac sodium gel (1%) using rat paw edema method (Figure 7). The developed ANE4-loaded chitosan gel caused 40.36 ± 2.02, 56.49 ± 1.86, 70.55 ± 0.95 and 90.79 ± 0.33% inhibition in rat paw edema at 1, 2, 3, and 4 h, respectively. However, Diclofenac sodium gel caused 29.56 ± 0.69, 50.59 ± 2.36, 63.65 ± 0.55 and 73.36 ± 1.61% inhibition at same period of treatment. The differences in the % inhibition by ANE4-loaded chitosan gel were statistically highly significant (*p* < 0.01) at 1, 3 and 4 h and statistically significant (*p* < 0.05) at 2 h. Based on data, it can be observed that the ANE4-loaded chitosan gel formulation exhibited higher anti-inflammatory activity in comparison to standard Diclofenac sodium gel, probably due better efficacy.

### 2.11. In-Vivo Wound Healing—Excision Model

**Figure 8:**
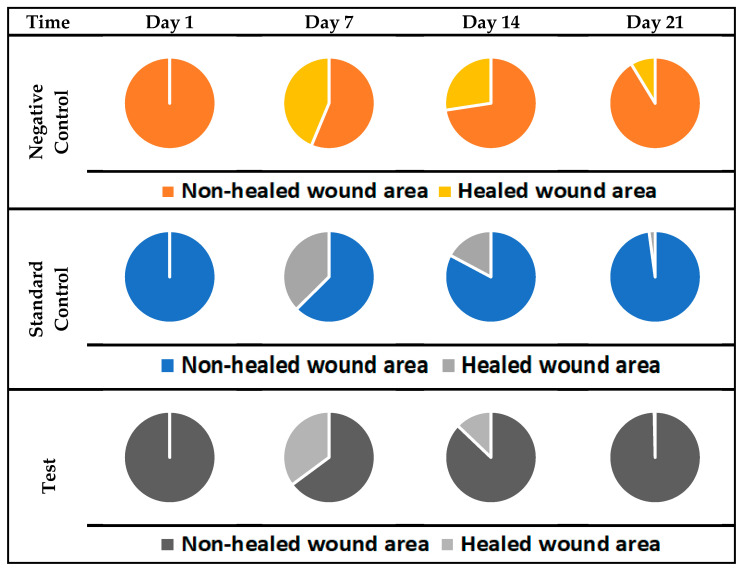
Wound sizes (mm)—fractions of wounds healed by different treatments at days 1, 7, 14 and 21 (*n* = 4 *p* < 0.05).

**Figure 9:**
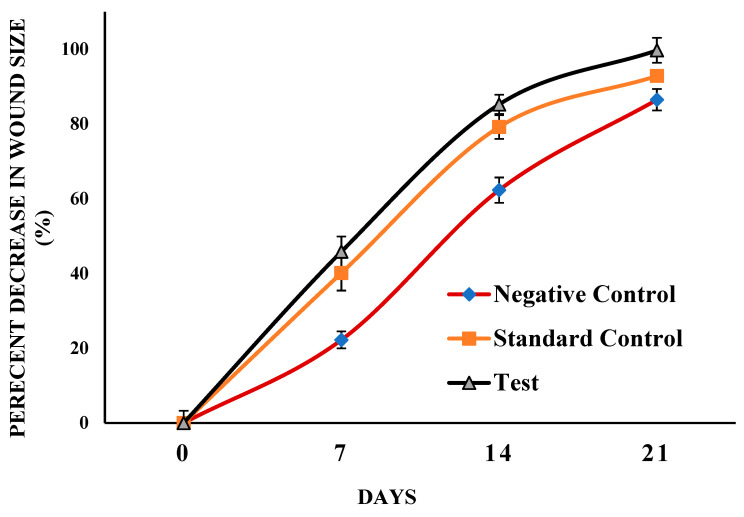
Decrease in wound sizes (%) wound healed by different treatments.at days 1, 7, 14 and 21 (Control, *n* = 4; STD, *n* = 4; TEST, *n* = 4; *p* < 0.05).

**Figure 10:**
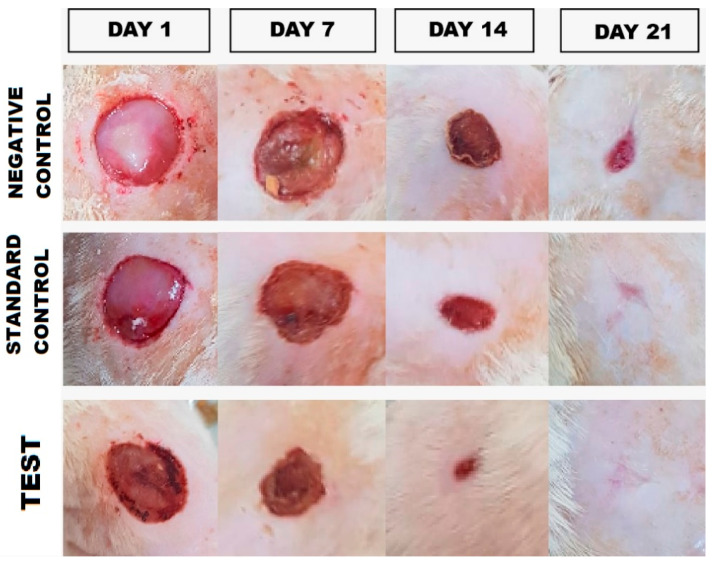
Photo images of the macroscopic observations of excision wound healed by different treatments at 1, 7, 14 and 21 days.

The excision model reflects the understanding of the wound healing process, wound size (mm) was measured, and results showed in Figure 8. For negative control, the average size was found to be (212.75 ± 5.35 mm), (165.5 ± 3.64 mm), (80.25 ± 1.47 mm), (20.25 ± 1.47 mm) after day 1, day 7, day 14, day 21, respectively. In standard control group (silver sulfadiazine cream), wound size at day 1 (241.25 ± 1.29 mm), day 7 (144.5 ± 3.50 mm) day 14 (50.25 ± 3.83 mm), and on day 21 (5.25 ± 0.43 mm) whereas for test (ANE4-loaded chitosan gel) the average wound measured was (240 ± 3.08 mm), (130 ± 1.58 mm), (35.5 ± 1.65 mm) (0.75 ± 0.82 mm) at 1, 7, 14 and 21 days. Moreover, the percentage of decreased in the wound size were calculated, the results, at 7th day was (22.20 ± 2.25%), (40.10 ± 3.26%), (45.82 ± 4.03%), at 14th day (62.27 ± 3.37%), (79.16 ± 4.69%), (85.21 ± 2.55%), and on 21st day (86.48 ± 2.87%), (92.82 ± 3.16%), (99.68 ± 3.34%) for the negative control, standard control and test treated group, respectively. As illustrated in the Figure of pie and curve Figure 9, treatment with ANE4-loaded chitosan gels decreased the wound size and increased the percentage of wound closure, which agrees with previous reports [32]. Figure 10, clearly depicts the progressive wound contraction caused by treatment with ANE4-loaded chitosan gels. Overall, our results showed an improved wound healing process with developed gels compared to the negative control and sulfadiazine treated standard control group.

### 2.12. Histopathology Study

**Figure 11:**
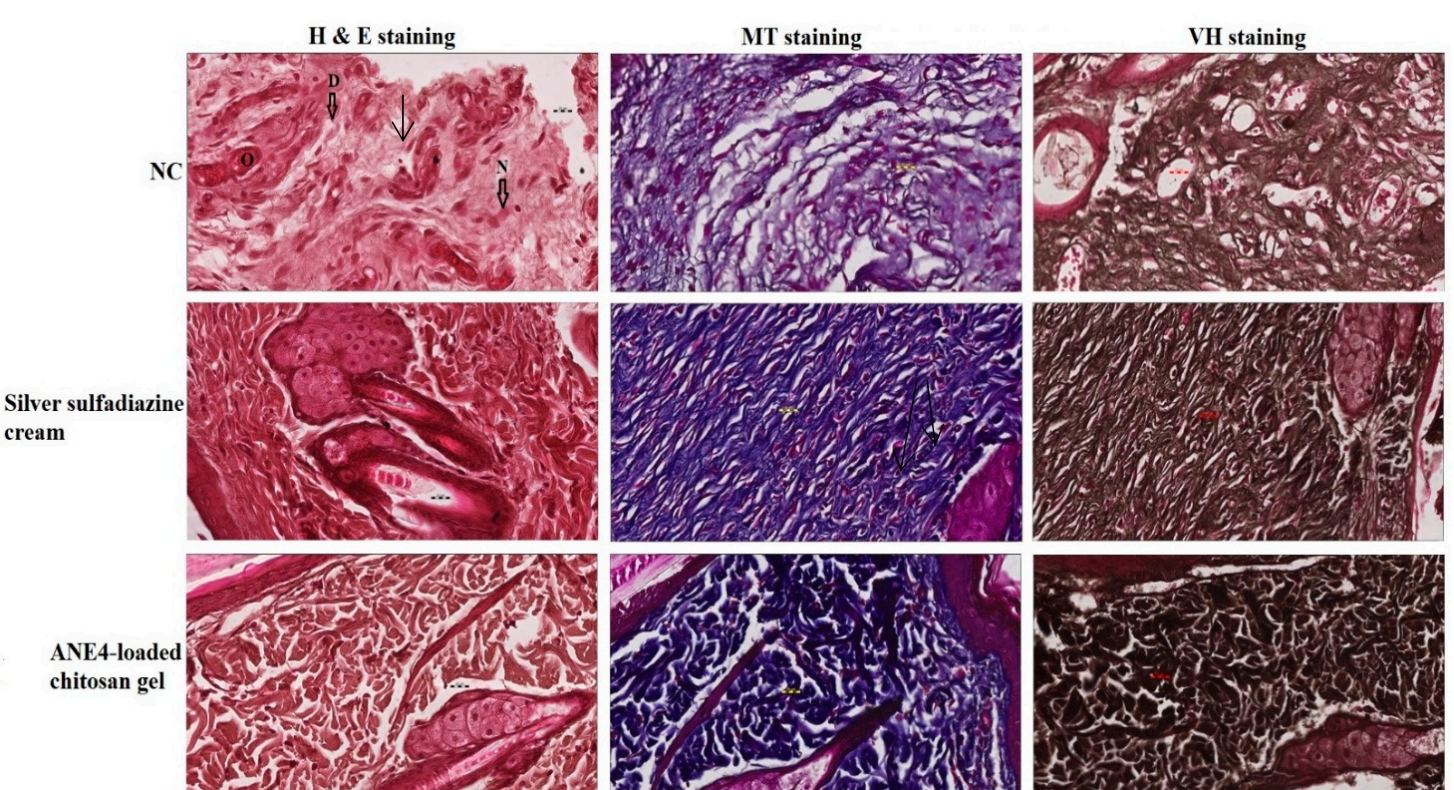
Photo images of skin samples after 21 days of treatment with ANE4-loaded chitosan gel and silver sulfadiazine cream with hematoxylin and eosin (H&E) stain, Masson Trichrome (MT) stain and Verhoeff hematoxylin (VH) stain (magnification 400×, and the scale bar is 20 µm). Negative control (NC) show severe tissue damage in the form of necrosis due to absence of nuclei as only remaining dissolved nuclei (N), degeneration in the form of vacuoles (D) as well as occlusion of a blood vessel by a large spot of hyaline material (O).

Negative control skin biopsy after H&E staining as well as Masson’s trichrome staining showed less volume of fibroblast and blood vessels compared to the standard control. However, the wound treated with the ANE4-loaded chitosan gel showed increased volume of fibroblasts and more regeneration of new blood vessels linked together with microvascular network. Inflammatory infiltration was more in the test, moderate in standard control-treated and lowered in the negative control, Figure 11. Masson’s trichrome negative control slides showed mild, thin (standard control) and thick collagen deposition at the wound center, Figure 11, respectively. In the test group skin tissue represents thicker collagen, well vascularization well stratified epidermis and hair follicles formation compared to the negative and standard controls. The inflammatory response was observed in negative control but relatively less in standard control and reduced in test specimen. Verhoeff special staining slides of Negative control (untreated) wound tissue showed in Figure 11, poor wound healing rate as indicated with the persistent inflammation marked by necrotic tissue and little proliferation at edges of a wound. Standard control (Silver sulphadiazine 1% cream) showed in Figure 11, marked re-epithelialization at wound edges indicates; granulation tissue formation evidenced by fibroblast proliferation and collagen synthesis. Figure 11, represents the wound healing activity, the center of the wound not fully healed. Test (ANE4-loaded chitosangels) treated wound tissue exhibits increased collagenation and re-epithelialization, indicative of accelerated wound healing rate (Figure 11). Histopathological study of exercised wound tissue showed improved wound healing rate indicated fibroblasts proliferation, neovascularization, epithelial regeneration, and collagen deposition activity in the test as compared to the negative (untreated) and standard control (Silver sulphadiazine 1% cream).

## 3. Conclusions

In this study, eucalyptus oil based NEs was developed as a nanocarrier; optimum APL-loaded NEs (APNE4) was then impregnated into chitosan gels for enhanced anti-inflammatory and wound healing activity. Optimized NEs (ANE4) containing EO (14%, *w*/*w*) as the oil phase and Tween-80 (20%, *w*/*w*), THP (40%, *w*/*w*) as surfactants and co-surfactants, respectively, were subjected to physicochemical characterization and pharmacological evaluations. In vitro release data showed 2.91 and 4.47 fold increase in APL release with sustained pattern from optimized NE (ANE4) and ANE4-loaded chitosan gel in comparison to pure APL drug suspension. Superior anti-inflammatory activity and improved wound healing efficiency were exhibited by ANE4-loaded chitosan gel in comparison with the negative and standard controls. This formulation is expected to improve therapeutic effectiveness and patient compliance with less systemic toxicity.

## 4. Materials and Methods

### 4.1. Materials

Apremilast (APL) was purchased from Beijing Mesochem Technology Co., Ltd., China. Kolliphore EL, Eucalyptus oil, Olive oil Tween 20 and Tween 80, Ethyl alcohol were procured from Sigma Aldrich, St. Louis, MO, USA. Lauroglycol 90, Capryol PGMC, and Transcutol HP were obtained from Gattefosse, Lyon, France. Ultrapure water collected from the Milli-Q Water purification system was used throughout the study. All the reagents and chemicals of analytical grades were used without any further purification and process.

### 4.2. Determination of APL Solubility in Oils, Surfactants, and Co-Surfactants

Solubility studies of APL were performed as described previously reported method [33] with some modification. Briefly, an excess amount of APL drug was added in 1.5 mL of oils (Kolliphore EL, Lauroglycol 90, Eucalyptus oil, Capryol PGMC and Olive oil), surfactant (Tween 20 and Tween 80), and co-surfactants (Ethyl alcohol and Transcutol HP) in a glass vial, and vials were tightly capped and kept on biological shaker “(LabTech, LBS-030S, Kyonggi, Korea)” for 72 h at 37 ± 2 °C. The samples were centrifuged at 6000 rpm “(HermleLabortechnik, Z216MK, Wehingen, Germany)” for 15 min. The supernatant was diluted with methanol appropriately and analyzed for drug content by UV/Vis spectrophotometer at wavelength 229 nm [27]. Based on the highest solubility, selected oil, surfactant, and co-surfactant were further used to construct the ternary phase diagram.

### 4.3. Pseudo-Ternary Phase Diagram

Based on solubility studies of APL in different components, distilled water (aqueous phase), Eucalyptus oil (oil), Tween-80 (surfactant), and THP (co-surfactant) were used for the construction of the ternary phase diagram. The surfactant and co-surfactant (Smix) at different weight ratios (1:0, 1:1, 1:2, and 2:1) were used to prepare the triple-phase diagram. Oil and Smix were thoroughly mixed at ratio 1:9 to 9:1 and titrated with distilled water till the formation of hazy dispersion [34,35].

### 4.4. Selection and Preparation of APL-Loaded NEs

Different formulations compositions were selected from the region of triple-phase diagrams to incorporate the APL drug (Table 3). Prepared nanoemulsion was enclosed in the screw vial and placed at room temperature to check the phase separation. Accurately weighed drug (1%, *w*/*w*) of the total weight of APL was dissolved in eucalyptus oil, vortexed (Vortex 3, IKA^®^-Werke GmbH & Co., Staufen, Germany) for 5 min, followed by addition of Smix composed of surfactant (Tween-80):co-surfactant (Transcutol HP), and stirred on a magnetic stirrer with a magnetic bar at room temperature until the dissolution of the drug. Then weighed amount of water (milli-Q) was added dropwise with continuous vortexing until isotropic bluish transparent systems were obtained, called NEs.

### 4.5. Physicochemical Properties of Developed APL-Loaded NEs

The developed NEs (ANE1-ANE5) were evaluated for mean droplet size, polydispersity index (PDI) and zeta potential (ZP) using “Malvern zetasizer (ZEN-3600, Malvern Instruments Ltd. Worcestershire, UK )”. The NEs sample was diluted with deionized water (1:200) and transferred into plastic cuvette in order to acquire dispersion, sonicated for 10 min to break the coalescence, then mean droplet size, PDI and ZP were measured by passing the laser at an angle of 90° at 25 °C operated by photon correlation spectroscopy [36,37]. The refractive index (RI) of APL-loaded NEs were determined using Abbe’s Refractometer “(Precision Testing Instruments Laboratory, Germany)” [38,39]. Percentage of transparency of the NEs was measured by homogenizing the sample with methanol, transmittance (%) was measured by UV- spectrophotometer at λmax 650 nm using methanol as blank [40].

### 4.6. Thermodynamic Stability Study

Developed APL-loaded NEs (ANE1-ANE5) were exposed to six cooling/heating cycles between 4 °C and 45 °C for 48 h. The NEs that did not show any phase separation, creaming, or cracking, were further tested for accelerated stability by keeping the sample to freeze-thaw cycles for 48 h at each temperature −21 and +25 °C [41]. NE was further subjected for centrifugation test by giving rotation at 3000 rpm for 20 min.

### 4.7. Morphology

The optimized NEs (ANE4) was diluted with milli-Q water (1:2 *v/v*), and coated on the carbon grid. The paper absorbed excess fluid, moisture also removed by placing it in the desiccator for about two hours before the analysis. Droplet morphologic characteristics were captured using JEM-1010 transmission electron microscope “(JEOL, Tokyo, Japan)” operated at 200 kV.

### 4.8. Drug Excipient Interaction FTIR Study

The FTIR spectrums of pure APL and optimized NES (APNE4) were taken by using FT-IR Spectrometer (Thermo Science iD5 ATR diamond Nicolet iS 5 FT-IR Spectrometer, Waltham, MA, USA). The samples were examined at the wavenumber range of 4000–400 cm^−1^ [42]. 

### 4.9. Formulation of NE-Loaded Gel

Chitosan gel (2%, *w/v*) was prepared by dispersing chitosan in distilled water. The polymeric dispersion was then kept in the refrigerator overnight after adding 2–3 drops of triethanolamine for soaking and swelling of the polymer. The optimized NE4 was incorporated into chitosan gel under magnetic stirring at a speed of 300 rpm for a 24 h at room temperature [43].

### 4.10. Evaluation of NE-Loaded Gel

#### 4.10.1. pH

Topical gel (APNE2) was dispersed into the purified water-dipped with the electrode. Hanna instrument (Hanna tools, Woonsocket, RI, USA) used to measure the pH was calibrated with the standard buffers; pH: 4.0, 7.0, and 9.0.

#### 4.10.2. Spreadability

The spreadability and rheological flow of gel is an essential parameter for patient-therapeutic compliance. The APNE2-loaded gel (0.5 gm) was placed between two plexus glass plates to measure spreadability. A circle (1 cm) marked on the external outer surface of plates, a weight of 500 gm was then placed on the top of the plate, and a sample was allowed to spread for 5 min. Increased in diameter due to the spreading was noted [44].

### 4.11. Diffusion Study and Release Kinetics Models

A diffusion study was carried out to plot the cumulative graphs and data utilized for release kinetics modeling. Franz diffusion cell of 10 mL capacity was used, filled with phosphate buffer (pH 7.4). The donor compartment was fixed with a dialysis membrane, molecular cut-off weight 14000 Da m (Sigma-Aldrich Co., St. Louis, MO, USA) and added with pure APL, optimized nanoemulsion (ANE4), and ANE4-loaded gel each containing 10 mg equivalent APL drug. The assembly was placed on a thermostatically controlled plate, acceptor compartment stirred by magnetic bead for 50 rpm maintained at 37 ± 0.5 °C [38]. At predetermined time intervals, samples were withdrawn, and the same volume was added with a fresh medium to replenish/sink condition. Samples were analyzed at 229 nm [27] using the UV-spectrophotometric method.

Release kinetics models; zero order, first order, Higuchi and Korsmeyer-Peppas model. Based on the regression analysis, the best model is proposed [35].

Q_t_ = Q_0_ + K_0_ t (1)

lnQ_t_ = lnQ_0_ + K_t_ (2)

(3) $$Q_{t}=K_{h}+\sqrt{t}$$

Q_t_ = K_KP_ t*^n^* (4)

Zero-order Equation (1). Q_t_ = amount of drug released at time t, Q_0_ = initial drug amount in solution, and K_0_ = Zero Order kinetic constant. First-order Equation (2). Q_t_ = amount of drug released at time t, Q_0_ = initial drug amount in solution, and K_t_ = First Order release constant. Higuchi Equation (3) and Korsmeyer Peppas model Equation (4). Where K is a kinetic constant and n is the drug release exponent. If *n* ≤ 0.45 then the drug release follows the Fickian diffusion (case I diffusional), 0.45 < *n* < 0.89 referred to anomalous (non-Fickian) diffusion.

### 4.12. Stability Study

APNE4 loaded chitosan gel was assessed for a stability of gel. Briefly, developed gel was kept at 25 ± 2 °C/75 ± 5% RH for six months, particle size and drug content were measured in order to check any significant change. Diffusion study was performed as per the above-mentioned procedure, release data then fitted to the similarity factor equation-5 and f2 value was noted. As per the SUPAC, if the f2 value ranges >50 [45], the release pattern is considered identical, and the product reflects stability.

(5) $$f_{2}=50\log\left\{{\left[1+\frac{1}{n}\sum_{t=1}^{n}w_{t}{\left(R_{t}-T_{t}\right)}^{2}\right]}^{-0.5}\right\}\times 100$$

where: *f*_2_ is a similarity factor; *n*, *w_t_*, *R_t_*, and *T_t_* are number of observations, optional weight, reference-APNE2 gel release (%), test-APNE2 gel- releases (%) after storage, respectively.

### 4.13. In-Vivo Animal Studies

#### 4.13.1. Animals

Healthy male Wistar rats (200–220 g) were received from the animal care unit of College of Pharmacy, Prince Sattam Bin Abdulaziz University, Alkharj, Saudi Arabia, and used to assess the Anti-inflammatory study and wound healing activity of gels. The rats were housed under standard laboratory of light and dark cycles, at 22 ± 2 °C and provided with standard diet. The Bioethical Research Committee approved the animal studies” (BERC-008–04-21)” Prince Sattam bin Abdulaziz University, Al-Kharj, Saudi Arabia.

#### 4.13.2. Anti-Inflammatory Study

Anti-inflammatory studies of prepared gel formulation was compared to fusidic acid gels using rat paw edema method [46]. Single dose parallel design was used for the anti-inflammatory studies. Twelve rats were randomly selected and divided into three group containing four animals (*n* = 4) each. Inflammation in the left hind paw of each rats were induced by injecting 0.1 mL (1%, *w/v*) carrageenan in normal saline. Animals in Group-I rats 1% CMC (NC) on pantar surface of left hind paw, rats of group II and group III received ANE4-loaded chitosan gel and Diclofenac sodium gel (1%), respectively. The rat paw volume of each rat was measured at 0, 1, 2, 3 and 4 h post injection of carrageenan using plethysmometer. The percent edema inhibition was calculated using following equation:

% Paw Edema Inhibition = (edema control rats-edema in treated rats)/(edema in control group) × 100

#### 4.13.3. Wound Healing—Excision Model

Developed ANE4-loaded gel was investigated for wound healing activity by excision models. All the rats grouped in three categories were anesthetized using ketamine (25 mg/kg) and xylazine (10 mg/kg), intraperitoneal injection. Hair clipper was used to shave the dorsal thoracic side, the cutaneous approximately circular wound of 230 mm width with 2 mm depth was inflicted using a surgical scissor. Rats were fed with the standard pellets and water ad libitum; they kept undressed to the open aseptic condition. The wound was then cleaned with normal saline (0.9% *w/v*, NaCl solution); after complete hemostasis, treatment was started; negative control (non-therapeutic), standard control (1% *w*/*w*, silver sulfadiazine cream) [47], test (ANE4-loaded chitosan gel) were applied to cover the wound every day until complete healing. Wound diameter was measured by using the OHP sheet at 1, 7, 14 and 21 days until complete epithelialization took place and with resulted in wound closure [48,49]. The data from all the groups were assessed to take the mean and Standard deviations. Results were used to calculate the area of the wound, wound healing, and percent decrease in the wound size.

Wound contraction (%) was calculated using the following equation.

% Wound Contraction = (Wound Area on day 0 − Wound Area on day n)/(Wound Area on day 0) × 100

#### 4.13.4. Histopathology Study

Skin tissue section of (3–5 cm) from three animal groups (negative control, standard control, and test) were collected into a suitable plastic closed container filled with 50 mL of 10% formalin (Sigma-Aldrich, MICH, USA) solution and processed in automated tissue processing machine (Leica ASP300S Fully Enclosed Tissue Processor, IL, USA). The processed tissue sample was embedded in paraffin, 5 µm thick section was prepared using a rotary microtome (SHUR/Cut 4500, TBS, NC, USA). Three sections from each group were stained; first section was stained with hematoxylin-eosin (H&E). The second and third sections were stained with masson trichrome (MT) and Verhoeff’s Hematoxylin (VH), respectively. Wound healing was observed under the microscope by expert histopathologists.

### 4.14. Statistical Analysis

“One-way ANOVA using Dunnett’s test. However, an unpaired *t*-test was used for the statistical evaluation. The GraphPad InStat software (GraphPad Software, San Diego, CA, USA) was used for statistical analysis, and *p* < 0.05 and *p* < 0.01 was considered significant and highly significant respectively”.

## Figures and Tables

**Table 1:** Physico-chemical evaluation of APL-loaded NEs (ANE1-ANE5).

NEs	Droplet Size (nm)	PDI	ZP (mV)	RI	% T
ANE1	23.07 ± 1.9	0.340 ± 0.003	−4.58 ± 1.31	1.311 ± 0.05	98.85 ± 0.82
ANE2	14.92 ± 1.1	0.078 ± 0.004	−21.35 ± 4.65	1.387 ± 0.03	97.91 ± 0.72
ANE3	196.6 ± 7.4	0.318 ± 0.038	−13.85 ± 2.75	1.373 ± 0.07	98.17 ± 0.96
ANE4	12.63 ± 1.2	0.269 ± 0.012	−23.00 ± 5.86	1.315 ± 0.02	99.89 ± 0.38
ANE5	50.4 ± 3.2	0.129 ± 0.029	−1.35 ± 0.00	1.421 ± 0.04	96.75 ± 0.16

**Table 2:** Drug release kinetic models.

Kinetic Models	R^2^	
Zero Order	0.6162	
First Order	0.8265	
Higuchi	0.8572	
Korsmayer-Peppas	0.9242	*n* = 0.2777

**Table 3:** Composition of APL-loaded nanoemulsions.

NEs Codes	Compositions (%*w*/*w*)				Smix
	EO	Tween^®^-80	THP^®^	Water	
ANE1	7	34	34	25	1:1
ANE2	18	36	36	10	1:1
ANE3	10	27	54	9	1:2
ANE4	14	20	40	26	1:2
ANE5	22	16	32	30	1:2

Each ml of NEs contains 1%, *w*/*w* of APL.

## Data Availability

The data presented in this study are available on request from the corresponding author.
